# Structural and electronic characteristic dataset of the water on basal surface the cis- and trans-vacant variety of a montmorillonite

**DOI:** 10.1016/j.dib.2023.109668

**Published:** 2023-10-11

**Authors:** Anton Kasprzhitskii, Alexander Kruglikov, Yakov Ermolov, Georgy Lazorenko

**Affiliations:** a Rostov State Transport University, Narodnogo Opolcheniya Sq., Rostov-on-Don, Russia; b Platov South-Russian State Polytechnic University, Prosveshcheniya Street, 132, Rostov Region, Novocherkassk, 346428, Russia; c Novosibirsk State University, Pirogov Street, 2, Novosibirsk, 630090, Russia

**Keywords:** DFT, Bound water, Montmorillonite, MMT, Octahedral substitution, OH-groups, Trans-vacant site, Cis-vacant site

## Abstract

The data given in the paper were obtained using CASTEP based on the density functional theory (DFT) applying a basis set of plane waves and PBE exchange-correlation functional. Van der Waals interactions were considered by the Grimme-D2 semi-empirical correction. The data include the optimized geometry and electronic properties of the equilibrium state of the non-hydrated *cis*- and *trans*-vacant variety of a Na-montmorillonite (MMT) and its state after the adsorption of water molecules. The data on hydration shells formed by the Na^+^ cation on the basal surface of MMT are also presented. The data are presented on the behavior of crystalline hydroxyl groups and water molecules during their adsorption. Data files of the optimized crystal structures and electronic properties can be read by the public text editors.

Specifications Table

**Table:** 

Subject	Surfaces and Interfaces
Specific subject area	Surface and Interface Chemistry of Clay Minerals
Type of data	Graph Figure
How the data were acquired	Sampling of the initial configurations of the adsorbed molecules on the montmorillonite surface was performed using Monte Carlo method implemented in the Adsorption Locator module of Material Studio software. The most stable states with the minimum system energy were further optimized. The final equilibrium geometry of the non-hydrated crystal structure of montmorillonite and its structure after the adsorption of water molecules on the basal surface, as well as their electronic properties, were obtained using CASTEP program.
Data format	Raw Analyzed
Description of data collection	The data were obtained using CASTEP program (version 2014) by employing the equipment of the shared research facilities of HPC computing resources at Lomonosov Moscow State University. Computational data (extension *.castep) is read by standard test data visualization programs, such as Notepad.
Data source location	*·Institution: Rostov State Transport University* *·City/Town/Region: Rostov-on-Don* *·Country: Russia*
Data accessibility	Repository name: Mendeley Data Data identification number: 10.17632/4d5yr7549k.1 Direct URL to data: https://data.mendeley.com/datasets/4d5yr7549k/1

## 1 Value of the Data


 

• The data contains structural and electronic characteristics of cis- and trans-vacant montmorillonite with a single isomorphic substitution Mg^2+^ → Al^3+^ in the octahedral position.
• It can be used to build scale models for solving tasks related to the interface phenomena. They make it possible to study the effect of the bound water on adsorption processes and contact phenomena.
• Data on the electronic structure (partial density of then electronic states) of the models makes it possible to evaluate changes in the state of the system atoms, related to the adsorption processes.

## 2 Objective

The models and related data were obtained during a theoretical study of the role of OH-crystalline groups in *cis*- and *trans*-vacant positions in the montmorillonite octahedral layer during the adsorption of water molecules. The obtained data were used to interpret the changes caused by the adsorption of water molecules on the basal surface of montmorillonite.

## 3 Data Description

**Fig. 1:**
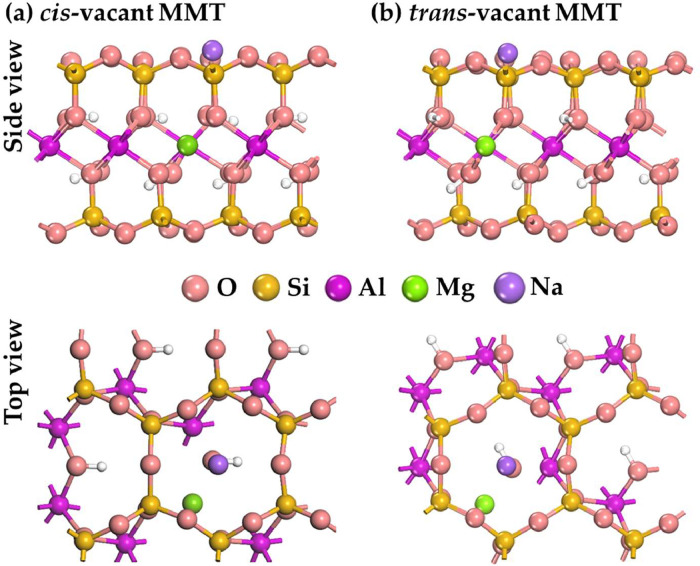
DFT optimized model of non-hydrated (a) *cis*- and (b) *trans*-vacant montmorillonite with single Mg^2+^ → Al^3+^ substitution in the octahedral position and Na^+^ cation.

Fig. 1 shows non-hydrated crystal structures of montmorillonite with a single isomorphic substitution Mg^2+^ → Al^3+^ in the octahedral position with *cis*- and *trans-*vacant states. Na^+^ cation was used to compensate the charge, appeared in the structure due to the isomorphic substitution.

The given geometric structures of montmorillonite (MMT) are optimized to achieve an equilibrium state. The dispersion forces were taken into account as a correction to the calculated total system energy. Consideration of this correction was required for the correct description of the crystalline OH-groups behavior in the octahedral MMT layers. The considered MMT models differ in the nature of the distribution of OH groups in the *cis-* and *trans*-vacant states of the octahedral layer.

**Fig. 2:**
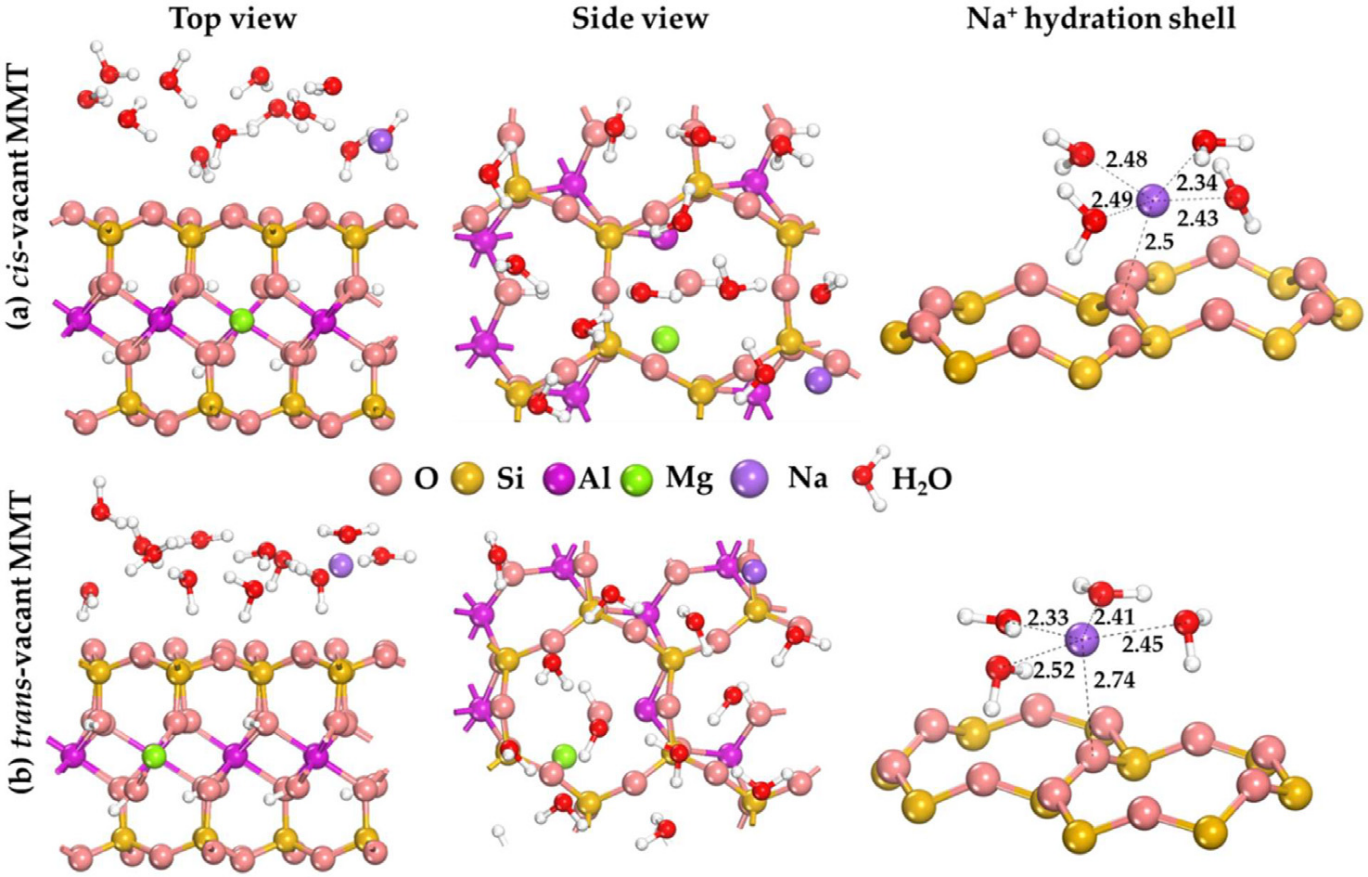
DFT optimized model of (a) *cis-* and (b) *trans-*vacant montmorillonite and 12 water molecules adsorbed on the basal surface. The distance values are shown in Å.

The data presented in Fig. 2 characterize the bound water layer formed on the basal surface of MMT for cis- and trans-vacant states. To obtain a layer of the adsorbed molecules, a preliminary sample of possible states with the lowest total energy was performed with Monte Carlo method. For the selected states, geometric optimization was performed using DFT method. The data is presented on the hydration shells formed around Na^+^ cation with the indicated interatomic distances.

**Fig. 3:**
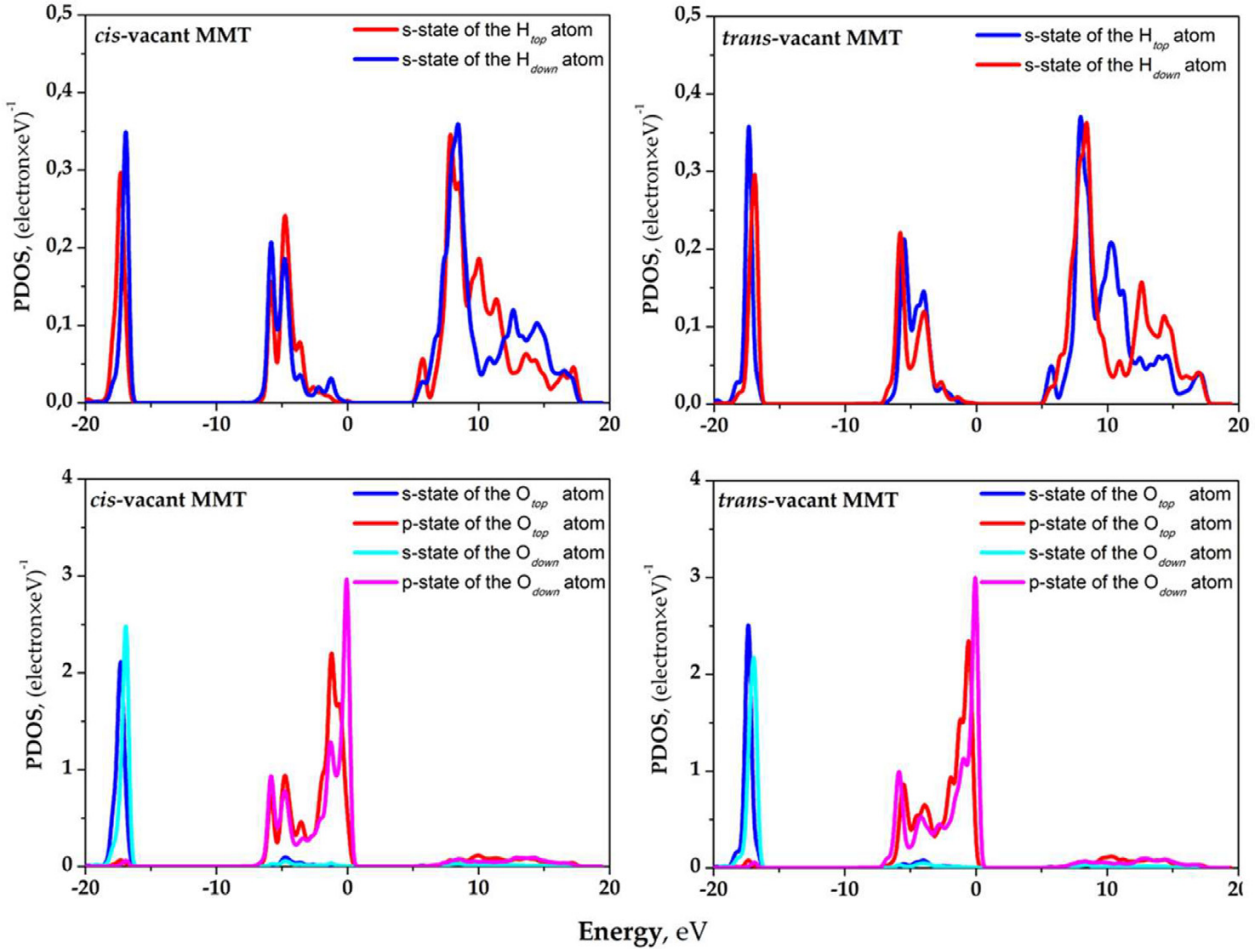
PDOS of OH-groups atoms of *cis*- and *trans*-vacant montmorillonite with a single Mg^2+^ → Al^3+^ substitution in the octahedral position.

**Fig. 4:**
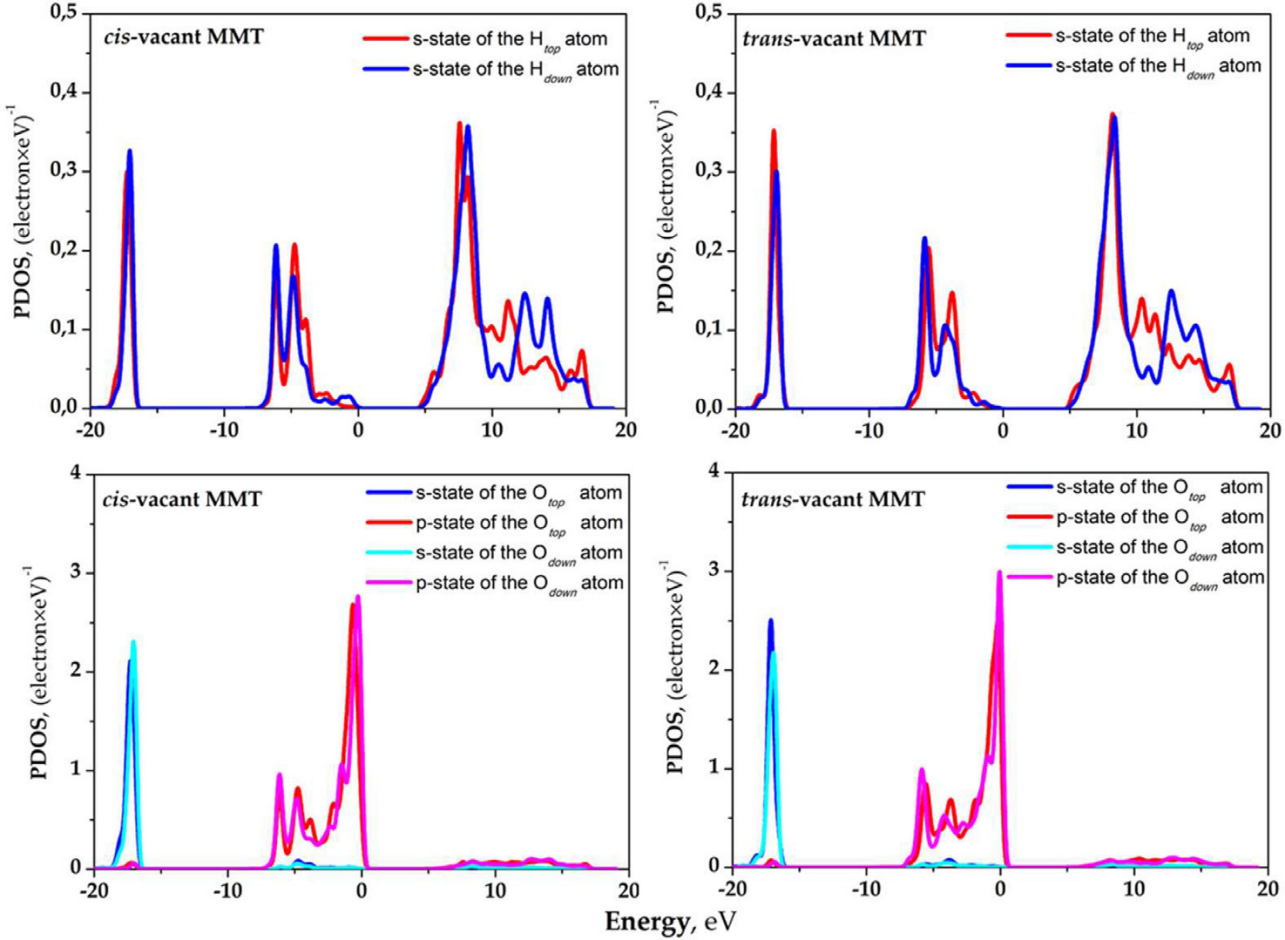
PDOS of OH-groups atoms of *cis*- and *trans*-vacant montmorillonite with the water molecules adsorbed on the basal surface.

**Fig. 5:**
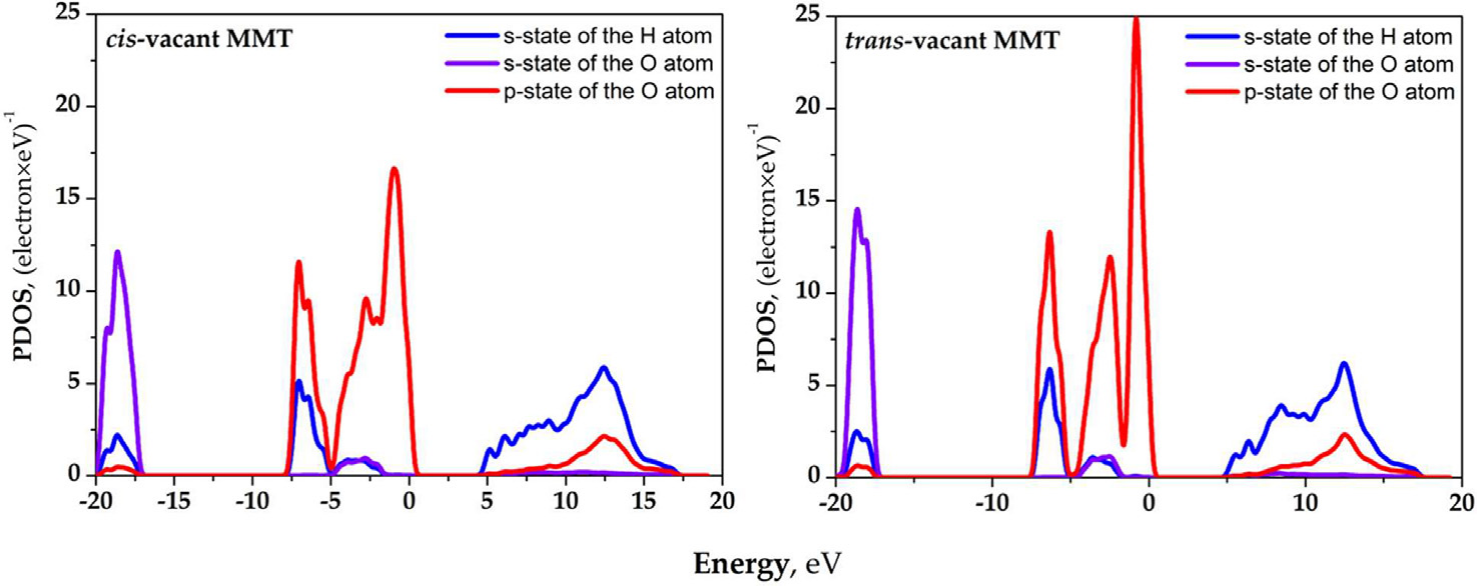
PDOS of atoms of water molecules adsorbed on the basal surface of *cis*- and *trans*-vacant montmorillonite with a single substitution Mg^2+^ → Al^3+^ in the octahedral position.

The partial densities of the electronic states (PDOS) of crystalline OH groups that characterize non-hydrated MMT models, models with an adsorbed layer of the water molecules, and the absorption water layer itself are shown in Fig. 3-5. Fig. 3 shows PDOS of the H and O atoms included in the upper and lower crystalline OH groups with respect to the MMT basal surface in the area of isomorphic Mg^2+^ → Al^3+^ substitution. Fig. 4 shows PDOS of crystalline OH group atoms that allow evaluating the changes related to the adsorption of the water molecules on the basal surface of MMT. Fig. 5 shows PDOS data for H and O atoms of the adsorbed water molecules. The obtained data make it possible to evaluate the changes connected with the process of adsorption of the water molecules on the basal surface of MMT.

## 4 Experimental Design, Materials and Methods

The non-hydrated model of the montmorillonite was created on the basis of two primitive unit cells forming a Si_16_Al_7_Mg_1_O_40_(OH)_8_Na supercell of 81 atoms with the sizes 10.36×8.89×15 Å. Montmorillonite with an adsorption layer of the water molecules consisting of 12 water molecules has the following configuration: Si_16_Al_7_Mg_1_O_40_(OH)_8_Na12(H_2_O).

The preliminary structure of the adsorption layer of the water molecules was obtained using Monte Carlo method implemented in the Adsorption Locator module of the Material Studio program [1]. The Metropolis algorithm with automatic temperature control was applied [2,3]. The calculation was done for 10 cycles with 100,000 steps in each cycle. The total energies calculation was fulfilled using ClayFF force field [4].

The optimized geometry and the electronic structure for all models were obtained using CASTEP software package [5]. DFT method was implemented in the package using the plane waves’ basis and periodic boundary conditions. The calculation was performed in PBE (Perdew-Burke-Ernzerhof) exchange-correlation potential [6]. Electronic correlations responsible for van der Waals interactions were considered by semi-empirical Grimm correction D2 [7]. To calculate the interaction of valence electrons 2s^2^2p^6^3s^1^ (Na), 2p^6^3s^2^ (Mg), 3s^2^3p^2^ (Si), 3s^2^3p^1^ (Al), 2s^2^2p^4^ (О) and 1s^1^ (H) with the ionic core, preliminarily generated ultra-soft pseudopotentials were used [8]. The cut-off energy of the basis set was chosen equal to 400 eV. Monkhorst-Pack 4 × 4 × 1 k-point grid [9] was chosen for the calculation. Geometry optimization was performed using Broyden-Fletcher-Goldfarb-Shanno procedure [10]. The following optimization parameters were used: total energy (10^−6^ eV/atom), maximum force (10^−2^ eV/Å), and maximum displacement (10^−4^ Å). The density of electronic states was calculated by the methods presented in [11].

## Limitations

Not applicable.

## Ethics Statements

This article does not contain any studies with human, animals subjects or any data collected from social media platforms. The datasets used in the article are open to the public. For the usage of these datasets, proper citation rules should be maintained.

## CRediT authorship contribution statement

**Anton Kasprzhitskii:** Conceptualization, Methodology, Validation. **Alexander Kruglikov:** Data curation, Writing – original draft. **Yakov Ermolov:** Visualization, Investigation. **Georgy Lazorenko:** Writing – review & editing.

## Data Availability

Structural and electronic characteristic dataset of the water on basal surface the cis- and trans-vacant variety of a montmorillonite (Original data) (Mendeley Data)

## References

[1] BIOVIA (2020).

[2] Metropolis N., Rosenbluth A.W., Rosenbluth M.N., Teller A.H., Teller E. (1953). Equation of state calculations by fast computing machines. J. Chem. Phys..

[3] Kirkpatrick S., Gelatt C.D., Vecchi M.P. (1983). Optimization by simulated annealing. Science.

[4] Cygan R.T., Greathouse J.A., Kalinichev A.G. (2021). Advances in Clayff molecular simulation of layered and nanoporous materials and their aqueous interfaces. J. Phys. Chem. C.

[5] Clark S.J., Segall M.D., Pickard C.J., Hasnip P.J., Probert M.I.J., Refson K., Payne M.C. (2005). First principles methods using CASTEP. Z. Kristallogr..

[6] Perdew J.P., Burke K., Ernzerhof M. (1996). Generalized gradient approximation made simple. Phys. Rev. Lett..

[7] Grimme S. (2006). Semiempirical GGA-type density functional constructed with a long-range dispersion correction. J. Comput. Chem..

[8] Vanderbilt D. (1990). Soft self-consistent pseudopotentials in a generalized eigenvalue formalism. Phys. Rev. B.

[9] Monkhorst H.J., Pack J.D. (1976). Special points for Brillouin-zone integrations. Phys. Rev. B.

[10] Pfrommer B.G., Côté M., Louie S.G., Cohen M.L. (1997). Relaxation of crystals with the Quasi-Newton Method. J. Comput. Phys..

[11] Sanchez-Portal D., Artacho E., Soler J.M. (1995). Projection of plane-wave calculations into atomic orbitals. Solid State Commun..

